# *N*-(Diphenyl­carbamo­yl)-*N*,*N*′,*N*′,*N*′′,*N*′′-penta­methyl­guanidinium tetra­phenyl­borate

**DOI:** 10.1107/S1600536812050507

**Published:** 2012-12-15

**Authors:** Ioannis Tiritiris

**Affiliations:** a Fakultät Chemie/Organische Chemie, Hochschule Aalen, Beethovenstrasse 1, D-73430 Aalen, Germany

## Abstract

In the title salt, C_19_H_25_N_4_O^+^·C_24_H_20_B^−^, the C=N and C—N bond lengths in the CN_3_ unit are 1.3327 (8)/1.3364 (9) and 1.3802 (9) Å, indicating double- and single-bond character, respectively. The N—C—N angles are 118.77 (6), 120.29 (6) and 120.81 (6)°, showing only a small deviation of the CN_3_ plane from an ideal trigonal-planar geometry. The bonds between the N atoms and the terminal methyl C atoms all have values close to a typical single bond [1.4636 (9)–1.4772 (9) Å]. The crystal packing is caused by electrostatic inter­actions between cations and anions.

## Related literature

For the synthesis and crystal structure of 3-[bis­(dimethyl­amino)­methyl­ene]-1,1-diphenyl­urea, see: Tiritiris (2012 ▶). For the crystal structures of alkali metal tetra­phenyl­borates, see: Behrens *et al.* (2012 ▶).
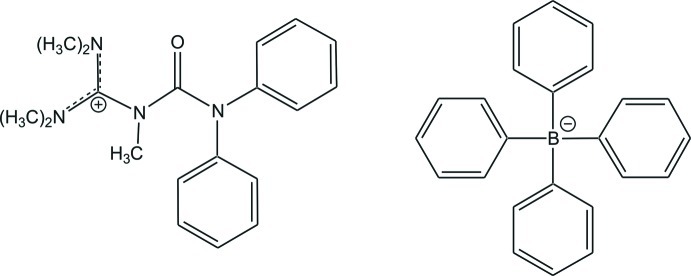


## Experimental

### 

#### Crystal data

C_19_H_25_N_4_O^+^·C_24_H_20_B^−^
*M* *_r_* = 644.64
Monoclinic, 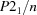

*a* = 11.0564 (4) Å
*b* = 9.5942 (3) Å
*c* = 33.4312 (12) Å
β = 91.684 (2)°
*V* = 3544.8 (2) Å^3^
*Z* = 4
Mo *K*α radiation
μ = 0.07 mm^−1^
*T* = 100 K
0.24 × 0.17 × 0.15 mm

#### Data collection

Bruker Kappa APEXII DUO diffractometer
119072 measured reflections
17178 independent reflections
14383 reflections with *I* > 2σ(*I*)
*R*_int_ = 0.026

#### Refinement

*R*[*F* ^2^ > 2σ(*F* ^2^)] = 0.043
*wR*(*F* ^2^) = 0.121
*S* = 1.06
17178 reflections
447 parameters
H-atom parameters constrained
Δρ_max_ = 0.51 e Å^−3^
Δρ_min_ = −0.22 e Å^−3^

### 

Data collection: *APEX2* (Bruker, 2008 ▶); cell refinement: *SAINT* (Bruker, 2008 ▶); data reduction: *SAINT*; program(s) used to solve structure: *SHELXS97* (Sheldrick, 2008 ▶); program(s) used to refine structure: *SHELXL97* (Sheldrick, 2008 ▶); molecular graphics: *DIAMOND* (Brandenburg & Putz, 2005 ▶); software used to prepare material for publication: *SHELXL97*.

## Supplementary Material

Crystal structure: contains datablock(s) I, global. DOI: 10.1107/S1600536812050507/fk2067sup1.cif

Structure factors: contains datablock(s) I. DOI: 10.1107/S1600536812050507/fk2067Isup2.hkl

Additional supplementary materials:  crystallographic information; 3D view; checkCIF report

## Figures and Tables

**Fig. 1.**
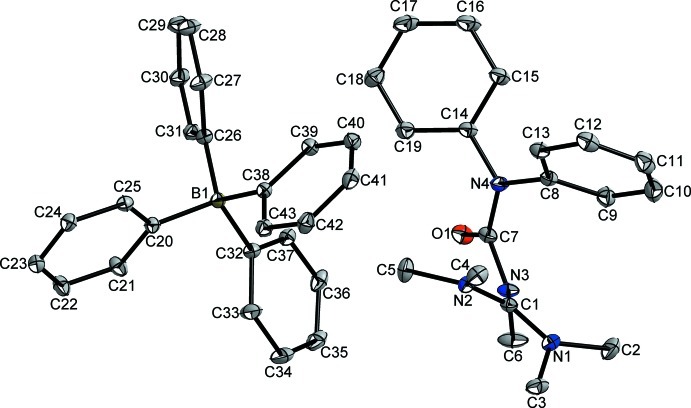
The molecular structure of the title compound with displacement ellipsoids drawn at the 50% probability level. H-atoms are omitted for the sake of clarity.

## supplementary crystallographic information

### Comment

3-[bis(dimethylamino)methylene]-1,1-diphenylurea - also known as *N*-diphenylcarbamoyl-*N*',*N*',*N*'',*N*''- tetramethylguanidine (Tiritiris, 2012) - is a guanidine derivative bearing additionally an urea moiety. By alkylation of the nitrogen atom, many structurally different guanidinium salts can be obtained. One of them is the here presented title compound. In the crystal structure of the salt, isolated cations and anions are present. One C–N bond of the CN_3_ unit in the cationic part is elongated (C1–N3 = 1.3802 (9) Å), indicating single bond character. The two remaining C–N bonds (C1–N2 = 1.3327 (8) Å and C1–N1 = 1.3364 (9) Å) are shorter and they show double bond character. As a consequence, the positive charge is not delocalized over the entire CN_3_ unit, but only between the two dimethylamino groups. The N–C1–N angles are: 120.29 (6)° (N1–C1–N2), 120.81 (6)° (N2–C1–N3) and 118.77 (6)° (N1–C1–N3), which indicate only a slight deviation of the CN_3_ plane from an ideal trigonal-planar geometry. The bonds between the N atoms and the terminal *C*-methyl groups, all have values close to a typical single bond (1.4636 (9)–1.4772 (9) Å). The C–O bond length in the diphenylcarbamoyl group is C7–O1 = 1.2148 (8) Å, and shows the expected double-bond character. The N–C bond lengths are: N3–C7 = 1.4267 (9) Å, N4–C7 = 1.3771 (8) Å, N4–C8 = 1.4348 (9) Å and N4–C14 = 1.4395 (9) Å. They are comparable with the data from the crystal structure analysis of 3-[bis(dimethylamino)methylene]- 1,1-diphenylurea (Tiritiris, 2012). The dihedral angle C1–N3–C7–N4 is 35.9 (1)° and the angle between the planes N1/C1/N2 and O1/C7/N4 is 61.5 (1)°, which show a significant twisting of the diphenylcarbamoyl group relative to the CN_3_ plane (Fig. 1). The bond lengths and angles in the tetraphenylborate ion are in good agreement with the data from the crystal structure analysis of the alkali metal tetraphenylborates (Behrens *et al.*, 2012). No specific interactions between the guanidinium ions and tetraphenylborate ions have been observed. Crystal packing is caused by electrostatic interactions between cations and anions.

### Experimental

The title compound was obtained by reacting one equivalent of 3-[bis(dimethylamino)methylene]-1,1-diphenylurea (Tiritiris, 2012) with one equivalent of dimethyl sulfate in acetonitrile for two hours at room temperature. After evaporation of the solvent, the remaining viscous mass is washed with diethyl ether and dried, giving *N*-diphenylcarbamoyl- *N*,*N*',*N*',*N*'',*N*''-pentamethylguanidinium methyl sulfate (I). To 1.0 g (2.29 mmol) of (I) in 20 ml acetonitrile, 0.78 g (2.29 mmol) of sodium tetraphenylborate in 20 ml acetonitrile was added. After fifteen minutes of stirring at room temperature, the precipitated sodium methyl sulfate was filtered off. The title compound crystallized from a saturated acetonitrile solution during storage for several days at 273 K, forming colorless single crystals. Yield: 1.3 g (88%). ^1^H NMR (500 MHz, CD_3_CN): δ = 2.83 [s, 12 H, N(CH_3_)_2_], 2.95 [s, 3 H, NCH_3_], 6.85–6.88 [t, J = 7 Hz, 4 H, C_6_H_5_], 7.06–7.09 [t, J = 6 Hz, 8 H, C_6_H_5_], 7.25–7.29 [m, 8 H, C_6_H_5_], 7.30–7.48 [m, 10 H, C_6_H_5_]. ^13^C NMR (125 MHz, CD_3_CN): δ = 38.0 (NCH_3_), 40.9 [N(CH_3_)_2_], 122.6 (C_6_H_5_), 125.4 (C_6_H_5_), 126.5–130.9 (C_6_H_5_), 136.5 (C_6_H_5_), 143.3 (C_6_H_5_), 156.8 (N_3_C^+^), 161.6 (C═O).

### Refinement

The hydrogen atoms of the methyl groups were derived from difference Fourier maps and allowed to rotate with a fixed angle around the C–N bond to best fit the experimental electron density, with *U*(H) set to 1.5 *U*_eq_(C) and d(C—H) = 0.98 Å. The H atoms in the aromatic rings were placed at calculated positions with (C—H) = 0.95 Å. They were included in the refinement in the riding model approximation, with *U*(H) set to 1.2 *U*_eq_(C).

### Crystal data

**Table:**

C_19_H_25_N_4_O^+^·C_24_H_20_B^−^	*F*(000) = 1376
*M**_r_* = 644.64	*D*_x_ = 1.208 Mg m^−^^3^
Monoclinic, *P*2_1_/*n*	Melting point: 499 K
Hall symbol: -P 2yn	Mo *K*α radiation, λ = 0.71073 Å
*a* = 11.0564 (4) Å	Cell parameters from 119072 reflections
*b* = 9.5942 (3) Å	θ = 1.9–36.3°
*c* = 33.4312 (12) Å	µ = 0.07 mm^−^^1^
β = 91.684 (2)°	*T* = 100 K
*V* = 3544.8 (2) Å^3^	Block, colourless
*Z* = 4	0.24 × 0.17 × 0.15 mm

### Data collection

**Table:**

Bruker Kappa APEXII DUO diffractometer	14383 reflections with *I* > 2σ(*I*)
Radiation source: sealed tube	*R*_int_ = 0.026
Graphite monochromator	θ_max_ = 36.3°, θ_min_ = 1.9°
φ scans, and ω scans	*h* = −18→18
119072 measured reflections	*k* = −15→15
17178 independent reflections	*l* = −49→55

### Refinement

**Table:**

Refinement on *F*^2^	Primary atom site location: structure-invariant direct methods
Least-squares matrix: full	Secondary atom site location: difference Fourier map
*R*[*F*^2^ > 2σ(*F*^2^)] = 0.043	Hydrogen site location: difference Fourier map
*wR*(*F*^2^) = 0.121	H-atom parameters constrained
*S* = 1.06	*w* = 1/[σ^2^(*F*_o_^2^) + (0.058*P*)^2^ + 0.8563*P*] where *P* = (*F*_o_^2^ + 2*F*_c_^2^)/3
17178 reflections	(Δ/σ)_max_ < 0.001
447 parameters	Δρ_max_ = 0.51 e Å^−^^3^
0 restraints	Δρ_min_ = −0.22 e Å^−^^3^

### Special details

**Table:**

Geometry. All e.s.d.'s (except the e.s.d. in the dihedral angle between two l.s. planes) are estimated using the full covariance matrix. The cell e.s.d.'s are taken into account individually in the estimation of e.s.d.'s in distances, angles and torsion angles; correlations between e.s.d.'s in cell parameters are only used when they are defined by crystal symmetry. An approximate (isotropic) treatment of cell e.s.d.'s is used for estimating e.s.d.'s involving l.s. planes.
Refinement. Refinement of *F*^2^ against ALL reflections. The weighted *R*-factor *wR* and goodness of fit *S* are based on *F*^2^, conventional *R*-factors *R* are based on *F*, with *F* set to zero for negative *F*^2^. The threshold expression of *F*^2^ > σ(*F*^2^) is used only for calculating *R*-factors(gt) *etc*. and is not relevant to the choice of reflections for refinement. *R*-factors based on *F*^2^ are statistically about twice as large as those based on *F*, and *R*- factors based on ALL data will be even larger.

### Fractional atomic coordinates and isotropic or equivalent isotropic displacement parameters (Å^2^)

**Table:**

	*x*	*y*	*z*	*U*_iso_*/*U*_eq_	
C1	1.00096 (5)	0.67518 (7)	0.146331 (19)	0.01387 (10)	
N1	1.07031 (5)	0.77454 (7)	0.163107 (18)	0.01749 (10)	
N2	0.88355 (5)	0.66826 (6)	0.154064 (17)	0.01423 (9)	
N3	1.05465 (5)	0.57515 (7)	0.123078 (17)	0.01599 (10)	
C2	1.18186 (7)	0.82630 (11)	0.14580 (3)	0.02881 (17)	
H2A	1.1908	0.7857	0.1192	0.043*	
H2B	1.2513	0.7998	0.1631	0.043*	
H2C	1.1781	0.9281	0.1436	0.043*	
C3	1.04672 (7)	0.82704 (8)	0.20332 (2)	0.02133 (13)	
H3A	1.0080	0.9187	0.2013	0.032*	
H3B	1.1233	0.8353	0.2187	0.032*	
H3C	0.9931	0.7622	0.2169	0.032*	
C4	0.81203 (6)	0.79278 (8)	0.16271 (2)	0.01889 (12)	
H4A	0.7991	0.7984	0.1915	0.028*	
H4B	0.7337	0.7872	0.1483	0.028*	
H4C	0.8555	0.8760	0.1540	0.028*	
C5	0.81622 (7)	0.53704 (8)	0.15524 (3)	0.02161 (13)	
H5A	0.8727	0.4586	0.1536	0.032*	
H5B	0.7580	0.5334	0.1326	0.032*	
H5C	0.7729	0.5313	0.1803	0.032*	
C6	1.16588 (7)	0.50748 (10)	0.13910 (2)	0.02623 (16)	
H6A	1.1864	0.5455	0.1657	0.039*	
H6B	1.2325	0.5253	0.1211	0.039*	
H6C	1.1525	0.4068	0.1412	0.039*	
C7	0.99758 (6)	0.50474 (7)	0.090013 (19)	0.01514 (10)	
O1	1.02242 (5)	0.38358 (6)	0.083615 (17)	0.02006 (10)	
N4	0.91755 (5)	0.58089 (6)	0.066452 (17)	0.01539 (9)	
C8	0.92431 (6)	0.72938 (7)	0.061944 (19)	0.01458 (10)	
C9	1.03421 (7)	0.79385 (8)	0.05482 (2)	0.02053 (12)	
H9A	1.1060	0.7400	0.0533	0.025*	
C10	1.03832 (8)	0.93813 (9)	0.04998 (3)	0.02628 (15)	
H10A	1.1138	0.9831	0.0464	0.032*	
C11	0.93289 (8)	1.01653 (8)	0.05035 (3)	0.02490 (14)	
H11A	0.9360	1.1148	0.0469	0.030*	
C12	0.82267 (7)	0.95055 (8)	0.05573 (2)	0.02075 (13)	
H12A	0.7500	1.0035	0.0550	0.025*	
C13	0.81810 (6)	0.80749 (7)	0.06212 (2)	0.01723 (11)	
H13A	0.7429	0.7632	0.0666	0.021*	
C14	0.83769 (6)	0.50707 (7)	0.039062 (19)	0.01551 (10)	
C15	0.82544 (7)	0.55132 (8)	−0.00044 (2)	0.01974 (12)	
H15A	0.8745	0.6247	−0.0100	0.024*	
C16	0.74043 (8)	0.48695 (9)	−0.02581 (2)	0.02552 (15)	
H16A	0.7303	0.5182	−0.0527	0.031*	
C17	0.67023 (8)	0.37747 (9)	−0.01230 (3)	0.02613 (15)	
H17A	0.6124	0.3343	−0.0298	0.031*	
C18	0.68496 (8)	0.33128 (9)	0.02700 (2)	0.02390 (14)	
H18A	0.6383	0.2552	0.0362	0.029*	
C19	0.76810 (7)	0.39665 (8)	0.05277 (2)	0.02067 (12)	
H19A	0.7775	0.3661	0.0797	0.025*	
B1	0.52389 (6)	0.21514 (8)	0.15651 (2)	0.01335 (11)	
C20	0.44464 (6)	0.14317 (7)	0.191805 (19)	0.01352 (10)	
C21	0.48685 (6)	0.03020 (7)	0.21503 (2)	0.01834 (11)	
H21A	0.5659	−0.0046	0.2108	0.022*	
C22	0.41710 (7)	−0.03286 (8)	0.24409 (2)	0.02053 (12)	
H22A	0.4494	−0.1085	0.2593	0.025*	
C23	0.30066 (6)	0.01450 (8)	0.25084 (2)	0.01820 (11)	
H23A	0.2530	−0.0278	0.2707	0.022*	
C24	0.25530 (6)	0.12485 (8)	0.22803 (2)	0.01747 (11)	
H24A	0.1758	0.1583	0.2322	0.021*	
C25	0.32589 (6)	0.18668 (7)	0.19911 (2)	0.01539 (10)	
H25A	0.2925	0.2612	0.1837	0.018*	
C26	0.47738 (5)	0.13982 (7)	0.114657 (19)	0.01352 (10)	
C27	0.37644 (6)	0.19096 (8)	0.09256 (2)	0.01912 (12)	
H27A	0.3359	0.2710	0.1022	0.023*	
C28	0.33342 (7)	0.12931 (8)	0.05717 (2)	0.02290 (14)	
H28A	0.2657	0.1684	0.0431	0.027*	
C29	0.38922 (7)	0.01102 (8)	0.04241 (2)	0.02233 (13)	
H29A	0.3613	−0.0305	0.0181	0.027*	
C30	0.48676 (7)	−0.04526 (8)	0.06403 (2)	0.01926 (12)	
H30A	0.5251	−0.1271	0.0547	0.023*	
C31	0.52867 (6)	0.01772 (7)	0.09943 (2)	0.01514 (10)	
H31A	0.5947	−0.0238	0.1138	0.018*	
C32	0.66957 (6)	0.19811 (7)	0.166723 (19)	0.01444 (10)	
C33	0.71593 (7)	0.22033 (10)	0.20592 (2)	0.02351 (14)	
H33A	0.6606	0.2388	0.2265	0.028*	
C34	0.83882 (7)	0.21646 (10)	0.21588 (3)	0.02745 (16)	
H34A	0.8658	0.2312	0.2428	0.033*	
C35	0.92237 (7)	0.19095 (8)	0.18636 (3)	0.02348 (14)	
H35A	1.0065	0.1879	0.1929	0.028*	
C36	0.88095 (6)	0.17006 (7)	0.14723 (2)	0.01941 (12)	
H36A	0.9369	0.1533	0.1267	0.023*	
C37	0.75681 (6)	0.17376 (7)	0.13797 (2)	0.01473 (10)	
H37A	0.7305	0.1591	0.1110	0.018*	
C38	0.50307 (5)	0.38509 (7)	0.154358 (18)	0.01357 (10)	
C39	0.51311 (6)	0.46073 (7)	0.11854 (2)	0.01621 (11)	
H39A	0.5259	0.4110	0.0945	0.019*	
C40	0.50512 (6)	0.60585 (8)	0.11693 (2)	0.01927 (12)	
H40A	0.5124	0.6525	0.0920	0.023*	
C41	0.48660 (6)	0.68271 (8)	0.15148 (2)	0.01973 (12)	
H41A	0.4806	0.7814	0.1505	0.024*	
C42	0.47705 (7)	0.61176 (8)	0.18754 (2)	0.01958 (12)	
H42A	0.4648	0.6624	0.2115	0.023*	
C43	0.48534 (6)	0.46658 (7)	0.18870 (2)	0.01693 (11)	
H43A	0.4787	0.4208	0.2137	0.020*	

### Atomic displacement parameters (Å^2^)

**Table:**

	*U*^11^	*U*^22^	*U*^33^	*U*^12^	*U*^13^	*U*^23^
C1	0.0132 (2)	0.0150 (2)	0.0133 (2)	0.00064 (18)	−0.00187 (17)	0.00090 (19)
N1	0.0149 (2)	0.0200 (3)	0.0174 (2)	−0.00287 (19)	−0.00295 (17)	−0.00106 (19)
N2	0.0131 (2)	0.0132 (2)	0.0164 (2)	−0.00042 (16)	0.00001 (16)	−0.00077 (17)
N3	0.0144 (2)	0.0196 (2)	0.0138 (2)	0.00508 (18)	−0.00286 (16)	−0.00122 (18)
C2	0.0179 (3)	0.0382 (5)	0.0302 (4)	−0.0107 (3)	−0.0011 (3)	−0.0005 (3)
C3	0.0237 (3)	0.0208 (3)	0.0191 (3)	−0.0004 (2)	−0.0063 (2)	−0.0046 (2)
C4	0.0165 (3)	0.0171 (3)	0.0230 (3)	0.0031 (2)	−0.0004 (2)	−0.0032 (2)
C5	0.0209 (3)	0.0161 (3)	0.0281 (3)	−0.0051 (2)	0.0047 (2)	−0.0016 (2)
C6	0.0216 (3)	0.0357 (4)	0.0209 (3)	0.0142 (3)	−0.0074 (2)	−0.0037 (3)
C7	0.0158 (2)	0.0164 (3)	0.0132 (2)	0.0025 (2)	−0.00034 (18)	0.00101 (19)
O1	0.0235 (2)	0.0166 (2)	0.0200 (2)	0.00525 (18)	−0.00059 (18)	−0.00018 (18)
N4	0.0178 (2)	0.0140 (2)	0.0140 (2)	0.00098 (18)	−0.00418 (17)	0.00090 (17)
C8	0.0161 (2)	0.0145 (2)	0.0130 (2)	0.00119 (19)	−0.00102 (18)	0.00093 (19)
C9	0.0174 (3)	0.0209 (3)	0.0233 (3)	−0.0002 (2)	0.0016 (2)	0.0040 (2)
C10	0.0266 (3)	0.0217 (3)	0.0307 (4)	−0.0053 (3)	0.0023 (3)	0.0055 (3)
C11	0.0355 (4)	0.0162 (3)	0.0231 (3)	−0.0004 (3)	0.0013 (3)	0.0021 (2)
C12	0.0274 (3)	0.0176 (3)	0.0173 (3)	0.0063 (2)	0.0005 (2)	−0.0003 (2)
C13	0.0177 (3)	0.0178 (3)	0.0161 (3)	0.0030 (2)	−0.0002 (2)	0.0002 (2)
C14	0.0182 (2)	0.0153 (3)	0.0129 (2)	0.0014 (2)	−0.00250 (19)	−0.00088 (19)
C15	0.0261 (3)	0.0198 (3)	0.0132 (3)	0.0008 (2)	−0.0019 (2)	0.0000 (2)
C16	0.0359 (4)	0.0254 (4)	0.0147 (3)	0.0010 (3)	−0.0078 (3)	−0.0018 (2)
C17	0.0305 (4)	0.0251 (4)	0.0222 (3)	−0.0008 (3)	−0.0096 (3)	−0.0062 (3)
C18	0.0278 (3)	0.0214 (3)	0.0222 (3)	−0.0051 (3)	−0.0043 (3)	−0.0036 (3)
C19	0.0268 (3)	0.0192 (3)	0.0158 (3)	−0.0040 (2)	−0.0033 (2)	0.0003 (2)
B1	0.0125 (2)	0.0153 (3)	0.0122 (3)	−0.0002 (2)	−0.00062 (19)	0.0003 (2)
C20	0.0141 (2)	0.0132 (2)	0.0132 (2)	−0.00016 (18)	−0.00034 (18)	0.00006 (18)
C21	0.0193 (3)	0.0166 (3)	0.0192 (3)	0.0037 (2)	0.0019 (2)	0.0039 (2)
C22	0.0242 (3)	0.0171 (3)	0.0203 (3)	0.0006 (2)	0.0009 (2)	0.0062 (2)
C23	0.0198 (3)	0.0191 (3)	0.0158 (3)	−0.0053 (2)	0.0003 (2)	0.0020 (2)
C24	0.0138 (2)	0.0214 (3)	0.0172 (3)	−0.0023 (2)	0.00034 (19)	0.0014 (2)
C25	0.0135 (2)	0.0167 (3)	0.0159 (3)	−0.00022 (19)	−0.00057 (18)	0.0022 (2)
C26	0.0129 (2)	0.0144 (2)	0.0132 (2)	−0.00080 (18)	−0.00111 (17)	0.00018 (19)
C27	0.0169 (3)	0.0185 (3)	0.0216 (3)	0.0026 (2)	−0.0068 (2)	−0.0036 (2)
C28	0.0241 (3)	0.0199 (3)	0.0239 (3)	0.0008 (2)	−0.0123 (3)	−0.0019 (2)
C29	0.0278 (3)	0.0194 (3)	0.0192 (3)	−0.0027 (3)	−0.0079 (2)	−0.0030 (2)
C30	0.0222 (3)	0.0150 (3)	0.0204 (3)	−0.0011 (2)	−0.0025 (2)	−0.0035 (2)
C31	0.0157 (2)	0.0131 (2)	0.0165 (3)	−0.00082 (19)	−0.00221 (19)	0.00027 (19)
C32	0.0132 (2)	0.0163 (3)	0.0137 (2)	−0.00047 (19)	−0.00181 (18)	0.00037 (19)
C33	0.0175 (3)	0.0376 (4)	0.0152 (3)	−0.0030 (3)	−0.0035 (2)	−0.0015 (3)
C34	0.0204 (3)	0.0393 (5)	0.0221 (3)	−0.0035 (3)	−0.0093 (2)	0.0016 (3)
C35	0.0147 (3)	0.0220 (3)	0.0333 (4)	0.0002 (2)	−0.0074 (2)	0.0004 (3)
C36	0.0132 (2)	0.0156 (3)	0.0293 (3)	0.0000 (2)	0.0001 (2)	−0.0025 (2)
C37	0.0134 (2)	0.0132 (2)	0.0176 (3)	−0.00071 (18)	−0.00013 (19)	−0.00067 (19)
C38	0.0125 (2)	0.0154 (2)	0.0127 (2)	−0.00173 (18)	−0.00025 (17)	0.00052 (19)
C39	0.0160 (2)	0.0181 (3)	0.0145 (2)	−0.0014 (2)	−0.00031 (19)	0.0023 (2)
C40	0.0174 (3)	0.0193 (3)	0.0210 (3)	−0.0024 (2)	−0.0019 (2)	0.0060 (2)
C41	0.0151 (2)	0.0153 (3)	0.0287 (3)	−0.0023 (2)	−0.0009 (2)	0.0019 (2)
C42	0.0198 (3)	0.0169 (3)	0.0222 (3)	−0.0034 (2)	0.0021 (2)	−0.0036 (2)
C43	0.0195 (3)	0.0161 (3)	0.0152 (3)	−0.0030 (2)	0.0013 (2)	−0.0007 (2)

### Geometric parameters (Å, º)

**Table:**

C1—N2	1.3327 (8)	C19—H19A	0.9500
C1—N1	1.3364 (9)	B1—C20	1.6424 (9)
C1—N3	1.3802 (9)	B1—C26	1.6437 (9)
N1—C2	1.4643 (10)	B1—C32	1.6447 (9)
N1—C3	1.4662 (10)	B1—C38	1.6480 (10)
N2—C5	1.4636 (9)	C20—C21	1.4050 (9)
N2—C4	1.4662 (9)	C20—C25	1.4059 (9)
N3—C7	1.4267 (9)	C21—C22	1.3960 (10)
N3—C6	1.4772 (9)	C21—H21A	0.9500
C2—H2A	0.9800	C22—C23	1.3898 (11)
C2—H2B	0.9800	C22—H22A	0.9500
C2—H2C	0.9800	C23—C24	1.3899 (10)
C3—H3A	0.9800	C23—H23A	0.9500
C3—H3B	0.9800	C24—C25	1.3929 (9)
C3—H3C	0.9800	C24—H24A	0.9500
C4—H4A	0.9800	C25—H25A	0.9500
C4—H4B	0.9800	C26—C31	1.4036 (9)
C4—H4C	0.9800	C26—C27	1.4083 (9)
C5—H5A	0.9800	C27—C28	1.3939 (10)
C5—H5B	0.9800	C27—H27A	0.9500
C5—H5C	0.9800	C28—C29	1.3893 (11)
C6—H6A	0.9800	C28—H28A	0.9500
C6—H6B	0.9800	C29—C30	1.3895 (11)
C6—H6C	0.9800	C29—H29A	0.9500
C7—O1	1.2148 (8)	C30—C31	1.3960 (10)
C7—N4	1.3771 (8)	C30—H30A	0.9500
N4—C8	1.4348 (9)	C31—H31A	0.9500
N4—C14	1.4395 (9)	C32—C37	1.4012 (9)
C8—C9	1.3903 (10)	C32—C33	1.4092 (10)
C8—C13	1.3932 (9)	C33—C34	1.3899 (11)
C9—C10	1.3945 (11)	C33—H33A	0.9500
C9—H9A	0.9500	C34—C35	1.3930 (13)
C10—C11	1.3877 (13)	C34—H34A	0.9500
C10—H10A	0.9500	C35—C36	1.3879 (11)
C11—C12	1.3896 (12)	C35—H35A	0.9500
C11—H11A	0.9500	C36—C37	1.3985 (9)
C12—C13	1.3903 (10)	C36—H36A	0.9500
C12—H12A	0.9500	C37—H37A	0.9500
C13—H13A	0.9500	C38—C43	1.4074 (9)
C14—C15	1.3902 (10)	C38—C39	1.4075 (9)
C14—C19	1.3947 (10)	C39—C40	1.3960 (10)
C15—C16	1.3918 (11)	C39—H39A	0.9500
C15—H15A	0.9500	C40—C41	1.3906 (11)
C16—C17	1.3894 (13)	C40—H40A	0.9500
C16—H16A	0.9500	C41—C42	1.3911 (11)
C17—C18	1.3915 (12)	C41—H41A	0.9500
C17—H17A	0.9500	C42—C43	1.3964 (10)
C18—C19	1.3906 (10)	C42—H42A	0.9500
C18—H18A	0.9500	C43—H43A	0.9500
			
N2—C1—N1	120.29 (6)	C18—C19—C14	120.00 (7)
N2—C1—N3	120.81 (6)	C18—C19—H19A	120.0
N1—C1—N3	118.77 (6)	C14—C19—H19A	120.0
C1—N1—C2	123.76 (6)	C20—B1—C26	105.46 (5)
C1—N1—C3	120.96 (6)	C20—B1—C32	110.42 (5)
C2—N1—C3	114.86 (6)	C26—B1—C32	114.39 (5)
C1—N2—C5	123.17 (6)	C20—B1—C38	111.75 (5)
C1—N2—C4	122.12 (6)	C26—B1—C38	110.98 (5)
C5—N2—C4	114.70 (6)	C32—B1—C38	104.01 (5)
C1—N3—C7	125.30 (5)	C21—C20—C25	115.45 (6)
C1—N3—C6	117.95 (6)	C21—C20—B1	123.08 (6)
C7—N3—C6	114.67 (6)	C25—C20—B1	121.37 (5)
N1—C2—H2A	109.5	C22—C21—C20	122.52 (6)
N1—C2—H2B	109.5	C22—C21—H21A	118.7
H2A—C2—H2B	109.5	C20—C21—H21A	118.7
N1—C2—H2C	109.5	C23—C22—C21	120.31 (6)
H2A—C2—H2C	109.5	C23—C22—H22A	119.8
H2B—C2—H2C	109.5	C21—C22—H22A	119.8
N1—C3—H3A	109.5	C22—C23—C24	118.75 (6)
N1—C3—H3B	109.5	C22—C23—H23A	120.6
H3A—C3—H3B	109.5	C24—C23—H23A	120.6
N1—C3—H3C	109.5	C23—C24—C25	120.32 (6)
H3A—C3—H3C	109.5	C23—C24—H24A	119.8
H3B—C3—H3C	109.5	C25—C24—H24A	119.8
N2—C4—H4A	109.5	C24—C25—C20	122.64 (6)
N2—C4—H4B	109.5	C24—C25—H25A	118.7
H4A—C4—H4B	109.5	C20—C25—H25A	118.7
N2—C4—H4C	109.5	C31—C26—C27	115.02 (6)
H4A—C4—H4C	109.5	C31—C26—B1	123.77 (5)
H4B—C4—H4C	109.5	C27—C26—B1	121.10 (6)
N2—C5—H5A	109.5	C28—C27—C26	122.88 (7)
N2—C5—H5B	109.5	C28—C27—H27A	118.6
H5A—C5—H5B	109.5	C26—C27—H27A	118.6
N2—C5—H5C	109.5	C29—C28—C27	120.27 (7)
H5A—C5—H5C	109.5	C29—C28—H28A	119.9
H5B—C5—H5C	109.5	C27—C28—H28A	119.9
N3—C6—H6A	109.5	C28—C29—C30	118.60 (7)
N3—C6—H6B	109.5	C28—C29—H29A	120.7
H6A—C6—H6B	109.5	C30—C29—H29A	120.7
N3—C6—H6C	109.5	C29—C30—C31	120.40 (7)
H6A—C6—H6C	109.5	C29—C30—H30A	119.8
H6B—C6—H6C	109.5	C31—C30—H30A	119.8
O1—C7—N4	123.49 (6)	C30—C31—C26	122.76 (6)
O1—C7—N3	119.50 (6)	C30—C31—H31A	118.6
N4—C7—N3	117.00 (6)	C26—C31—H31A	118.6
C7—N4—C8	123.53 (6)	C37—C32—C33	115.09 (6)
C7—N4—C14	118.33 (6)	C37—C32—B1	124.32 (6)
C8—N4—C14	117.04 (5)	C33—C32—B1	120.35 (6)
C9—C8—C13	120.16 (6)	C34—C33—C32	123.03 (7)
C9—C8—N4	120.55 (6)	C34—C33—H33A	118.5
C13—C8—N4	119.15 (6)	C32—C33—H33A	118.5
C8—C9—C10	119.55 (7)	C33—C34—C35	119.93 (7)
C8—C9—H9A	120.2	C33—C34—H34A	120.0
C10—C9—H9A	120.2	C35—C34—H34A	120.0
C11—C10—C9	120.44 (8)	C36—C35—C34	119.07 (7)
C11—C10—H10A	119.8	C36—C35—H35A	120.5
C9—C10—H10A	119.8	C34—C35—H35A	120.5
C10—C11—C12	119.63 (7)	C35—C36—C37	119.96 (7)
C10—C11—H11A	120.2	C35—C36—H36A	120.0
C12—C11—H11A	120.2	C37—C36—H36A	120.0
C11—C12—C13	120.37 (7)	C36—C37—C32	122.92 (6)
C11—C12—H12A	119.8	C36—C37—H37A	118.5
C13—C12—H12A	119.8	C32—C37—H37A	118.5
C12—C13—C8	119.73 (7)	C43—C38—C39	115.08 (6)
C12—C13—H13A	120.1	C43—C38—B1	122.46 (6)
C8—C13—H13A	120.1	C39—C38—B1	122.20 (6)
C15—C14—C19	120.32 (6)	C40—C39—C38	122.71 (6)
C15—C14—N4	119.66 (6)	C40—C39—H39A	118.6
C19—C14—N4	119.92 (6)	C38—C39—H39A	118.6
C14—C15—C16	119.24 (7)	C41—C40—C39	120.51 (7)
C14—C15—H15A	120.4	C41—C40—H40A	119.7
C16—C15—H15A	120.4	C39—C40—H40A	119.7
C17—C16—C15	120.75 (7)	C40—C41—C42	118.50 (7)
C17—C16—H16A	119.6	C40—C41—H41A	120.8
C15—C16—H16A	119.6	C42—C41—H41A	120.8
C16—C17—C18	119.77 (7)	C41—C42—C43	120.36 (7)
C16—C17—H17A	120.1	C41—C42—H42A	119.8
C18—C17—H17A	120.1	C43—C42—H42A	119.8
C19—C18—C17	119.89 (8)	C42—C43—C38	122.83 (6)
C19—C18—H18A	120.1	C42—C43—H43A	118.6
C17—C18—H18A	120.1	C38—C43—H43A	118.6
			
N2—C1—N1—C2	−154.84 (7)	C25—C20—C21—C22	−1.46 (10)
N3—C1—N1—C2	29.32 (10)	B1—C20—C21—C22	−177.94 (7)
N2—C1—N1—C3	33.03 (10)	C20—C21—C22—C23	0.59 (12)
N3—C1—N1—C3	−142.81 (7)	C21—C22—C23—C24	0.32 (11)
N1—C1—N2—C5	−147.91 (7)	C22—C23—C24—C25	−0.27 (11)
N3—C1—N2—C5	27.85 (10)	C23—C24—C25—C20	−0.70 (11)
N1—C1—N2—C4	31.14 (9)	C21—C20—C25—C24	1.52 (10)
N3—C1—N2—C4	−153.11 (6)	B1—C20—C25—C24	178.06 (6)
N2—C1—N3—C7	34.10 (10)	C20—B1—C26—C31	−89.37 (7)
N1—C1—N3—C7	−150.08 (7)	C32—B1—C26—C31	32.16 (9)
N2—C1—N3—C6	−128.56 (7)	C38—B1—C26—C31	149.45 (6)
N1—C1—N3—C6	47.25 (9)	C20—B1—C26—C27	86.83 (7)
C1—N3—C7—O1	−145.19 (7)	C32—B1—C26—C27	−151.64 (6)
C6—N3—C7—O1	17.98 (10)	C38—B1—C26—C27	−34.35 (8)
C1—N3—C7—N4	35.91 (10)	C31—C26—C27—C28	−2.83 (11)
C6—N3—C7—N4	−160.93 (7)	B1—C26—C27—C28	−179.34 (7)
O1—C7—N4—C8	−152.04 (7)	C26—C27—C28—C29	0.91 (13)
N3—C7—N4—C8	26.82 (9)	C27—C28—C29—C30	1.16 (13)
O1—C7—N4—C14	15.60 (10)	C28—C29—C30—C31	−1.13 (12)
N3—C7—N4—C14	−165.54 (6)	C29—C30—C31—C26	−0.98 (11)
C7—N4—C8—C9	46.21 (10)	C27—C26—C31—C30	2.86 (10)
C14—N4—C8—C9	−121.57 (7)	B1—C26—C31—C30	179.27 (6)
C7—N4—C8—C13	−138.05 (7)	C20—B1—C32—C37	143.05 (6)
C14—N4—C8—C13	54.16 (8)	C26—B1—C32—C37	24.28 (9)
C13—C8—C9—C10	3.42 (11)	C38—B1—C32—C37	−96.93 (7)
N4—C8—C9—C10	179.11 (7)	C20—B1—C32—C33	−42.78 (9)
C8—C9—C10—C11	−3.07 (13)	C26—B1—C32—C33	−161.54 (7)
C9—C10—C11—C12	0.25 (13)	C38—B1—C32—C33	77.25 (8)
C10—C11—C12—C13	2.24 (12)	C37—C32—C33—C34	−0.99 (12)
C11—C12—C13—C8	−1.89 (11)	B1—C32—C33—C34	−175.68 (8)
C9—C8—C13—C12	−0.96 (10)	C32—C33—C34—C35	0.61 (14)
N4—C8—C13—C12	−176.71 (6)	C33—C34—C35—C36	0.16 (13)
C7—N4—C14—C15	−132.19 (7)	C34—C35—C36—C37	−0.47 (12)
C8—N4—C14—C15	36.25 (9)	C35—C36—C37—C32	0.05 (11)
C7—N4—C14—C19	51.37 (9)	C33—C32—C37—C36	0.66 (10)
C8—N4—C14—C19	−140.18 (7)	B1—C32—C37—C36	175.11 (6)
C19—C14—C15—C16	1.91 (11)	C20—B1—C38—C43	35.08 (8)
N4—C14—C15—C16	−174.52 (7)	C26—B1—C38—C43	152.49 (6)
C14—C15—C16—C17	−1.50 (12)	C32—B1—C38—C43	−84.04 (7)
C15—C16—C17—C18	−0.08 (13)	C20—B1—C38—C39	−151.05 (6)
C16—C17—C18—C19	1.27 (13)	C26—B1—C38—C39	−33.64 (8)
C17—C18—C19—C14	−0.86 (13)	C32—B1—C38—C39	89.83 (7)
C15—C14—C19—C18	−0.74 (12)	C43—C38—C39—C40	−0.53 (9)
N4—C14—C19—C18	175.67 (7)	B1—C38—C39—C40	−174.82 (6)
C26—B1—C20—C21	95.42 (7)	C38—C39—C40—C41	0.05 (10)
C32—B1—C20—C21	−28.65 (9)	C39—C40—C41—C42	0.39 (10)
C38—B1—C20—C21	−143.90 (6)	C40—C41—C42—C43	−0.33 (10)
C26—B1—C20—C25	−80.85 (7)	C41—C42—C43—C38	−0.18 (11)
C32—B1—C20—C25	155.07 (6)	C39—C38—C43—C42	0.59 (10)
C38—B1—C20—C25	39.83 (8)	B1—C38—C43—C42	174.87 (6)
